# Armand Charles Goubaux

**Published:** 1890-08

**Authors:** 


### NECROLOGY.

### ARMAND CHARLES GOUBAUX.

ARMAND CHARLES GOUBAUX, Honorary Director of the Veterinary School at Alfort, France, died on June 29th, at St. Maurice, where he had lived since retiring from his active duties as Director of the Alfort School.

Monsieur Goubauk received his diploma in 1831, and re-entered the school at once as a teacher. He successively held the positions of Chief of Anatomy, Professor of Anatomy, Professor of Sanitary Police, and Director of the Alfort Veterinary School. He was a member of numerous scientific societies, and the author of many valuable veterinary works. But while his scientific work will honor him peimanently as one of the leaders of the veterinary profession, those who had the pleasure of obtaining instruction from him will remember him best as the most genial and kindest-hearted of teachers and friends.

